# Genotyping of Human *Brucella melitensis* Biovar 3 Isolated from Shanxi Province in China by MLVA16 and HOOF

**DOI:** 10.1371/journal.pone.0115932

**Published:** 2015-01-23

**Authors:** Pei Xiao, Hongxia Yang, Dongdong Di, Dongri Piao, Qiuxiang Zhang, Ruie Hao, Suxia Yao, Rong Zhao, Fanfei Zhang, Guozhong Tian, Hongyan Zhao, Weixing Fan, Buyun Cui, Hai Jiang

**Affiliations:** 1 State Key Laboratory for Infectious Disease Prevention and Control, Collaborative Innovation Center for Diagnosis and Treatment of Infectious Diseases, National Institute for Communicable Disease Control and Prevention, Chinese Center for Disease Control and Prevention, Beijing, China; 2 Disease Inspection Laboratory, Shanxi Center for Disease Control and Prevention, Taiyuan, China; 3 Laboratory of Zoonoses, China Animal Health and Epidemiology Center, MOA, Qingdao, China; University of Minnesota, UNITED STATES

## Abstract

**Background:**

Brucellosis presents a significant economic burden for China because it causes reproductive failure in host species and chronic health problems in humans. These problems can involve multiple organs. Brucellosis is highly endemic in Shanxi Province China. Molecular typing would be very useful to epidemiological surveillance. The purpose of this study was to assess the diversity of *Brucella melitensis* strains for epidemiological surveillance. Historical monitoring data suggest that *Brucella melitensis* biovar 3 is the predominant strain associated with the epidemic of brucellosis in Shanxi Province.

**Methods/Principal Findings:**

Multiple-locus variable-number repeat analysis (MLVA-16) and hypervariable octameric oligonucleotide fingerprinting (HOOF-print) were used to type a human-hosted *Brucella melitensis* population (81 strains). Sixty-two MLVA genotypes (discriminatory index: 0.99) were detected, and they had a genetic similarity coefficient ranging from 84.9% to 100%. Eighty strains of the population belonged to the eastern Mediterranean group with panel 1 genotypes 42 (79 strains) and 43 (1 strain). A new panel 1 genotype was found in this study. It was named 114 MLVAorsay genotype and it showed similarity to the two isolates from Guangdong in a previous study. *Brucella melitensis* is distributed throughout Shanxi Province, and like samples from Inner Mongolia, the eastern Mediterranean genotype 42 was the main epidemic strain (97%). The HOOF-printing showed a higher diversity than MLVA-16 with a genetic similarity coefficient ranging from 56.8% to 100%.

**Conclusions:**

According to the MLVA-16 and HOOF-printing results, both methods could be used for the epidemiological surveillance of brucellosis. A new genotype was found in both Shanxi and Guangdong Provinces. In areas with brucellosis, the MLVA-16 scheme is very important for tracing cases back to their origins during outbreak investigations. It may facilitate the expansion and eradication of the disease.

## Introduction

Brucellosis is one of the most common anthropozoonoses worldwide [1,2]. The disease is usually transmitted from animal reservoirs of *Brucella spp*. to humans by direct contact with infected animals or through ingestion of raw milk or unpasteurized cheese [3]. Brucellosis presents a significant economic burden because it causes reproductive failure in host species and chronic health problems in humans. These problems can involve multiple organs [4]. Although it is controlled in many industrialized countries, the disease remains endemic in many parts of the world, including Spain, the Middle East, Latin America, and Asia [5]. In developing countries, most human cases are caused by *B. melitensis*, particularly by its biovars 1 and 3 [6,7]. Recent studies have confirmed that both hypervariable oligonucleotide finger printing (HOOF-prints) and multilocus variable-number tandem-repeat analysis (MLVA-16), which are based on tandem repeats, are useful for epidemiological surveillance and genotyping of *Brucella* strains [8–11]. In China, suspected and confirmed cases of brucellosis must be reported to the local and provincial Centers for Disease Control and Prevention (CDC) and then to the national CDC through the National Notifiable Disease Surveillance System [2]. The incidence of human brucellosis has increased rapidly over the past five years. Brucellosis remains a serious public health issue in the northern China, where people are economically dependent on ruminant livestock. Shanxi Province is in northern China, and it accounts for approximately 60% of the area’s agricultural population. Among Chinese provinces, Shanxi has the third-highest incidence of brucellosis. Data from the National Notifiable Disease Surveillance System show that a total of 13,791 brucellosis cases occurred in a Shanxi’s population of 35.93 million between 2009 and 2011. In this study, both MLVA-16 and HOOF were used to characterize *B. melitensis* biovar 3 strains. These strains were isolated at the sentinel sites in Shanxi Province, China, between 2009 and 2011. The purpose of this study was to evaluate the resolution of MLVA-16 and HOOF and to assess the diversity of *Brucella melitensis* strains for epidemiological surveillance.

## Materials and Methods

### Ethics statement

This study is a retrospective investigation of our institute’s strain collection. It was performed using modern typing methods. After approval by the ethics committee, we included patient data in this study (Ethics Committee, National Institute for Communicable Disease Control and Prevention, Chinese Center for Disease Control and Prevention).

### Bacterial strains

There were 22, 35, and 39 sentinel counties in Shanxi Province in 2009, 2010, and 2011, respectively. All 81 strains (3 from 2009, 30 from 2010, and 48 from 2011) were collected from 24 of these sentinel counties between 2009 and 2011. The *Brucella* bacteria were isolated using standard methods [12]. All 81 strains were identified as *Brucella spp.* and analyzed using conventional biotyping methods, including the CO_2_ requirements, H_2_S production, urease activity, sensitivity to thionin and basic fuchsin, phage sensitivity, and agglutination with monospecific A and M antisera [12]. Whole genomic DNA was extracted with a DNeasy Blood and Tissue Kit (Qiagen China Ltd., China) by following the manufacturer’s protocol for extraction of genomic DNA from gram-negative bacteria. The AMOS PCR was used to confirm the species of the strains using the five-primer cocktails targeting the IS711 sequence [13].

### Scheme of MLVA-16 and HOOF-print genotyping

Sixteen tandem-repeat loci of MLVA were identified in the 81 strains, using the previously described methods [8, 9]. These loci were divided into three groups as previously described [8]: panel 1 (8 loci, including bruce06, bruce08, bruce11, bruce12, bruce42, bruce43, bruce45, and bruce55), panel 2A (3 loci, including bruce18, bruce19, and bruce21), and panel 2B (5 loci, including bruce04, bruce07, bruce09, bruce16, and bruce30). All strains were also typed using a previously described method for HOOF-print [10, 11]. The bruce04, bruce09, and bruce30 markers of the MLVA-16 were found to be equivalent to tandem repeats (TR) 6, TR8, and TR2 in HOOF-print analysis. Five microliters of the amplification products were loaded on 2% or 3% agarose gels with ethidium bromide (0.5 μg/ml), visualized under UV light, and photographed. PCR products were purified and directly sequenced, using an ABI Prism Big Dye Terminator (v3.1) cycle sequencing ready reaction kit (v5.0).

### Data analysis

The number of repeats for MLVA-16 and HOOF-print typing was estimated by comparing the molecular sizes and sequences of the known oligonucleotides to those of *B. melitensis* 16 M. The genotypes of the MLVA-16 and HOOF-print were determined by combining the allelic profiles of the markers. Fragment sizes were converted to tandem repeat unit (U) numbers and imported into BioNumerics v4.0 (Applied Maths, Belgium) as a character data set. Clustering analyses used the categorical coefficient and the UPGMA (unweighted pair group method using arithmetic averages). Not all loci evolved at the same rate. Genetic diversity was then calculated (Hunter-Gaston diversity index [HGDI]) (http://www.hpa-bioinformatics.org.uk/cgi-bin/DICI/DICI.pl). Values of the HGDI can range from 0.0 (no diversity) to 1.0 (complete diversity) [14, 15]. The genotyping data of MLVA-16 and HOOF-print can be found in the supplementary data (S1 Table and S2 Table). MLVA genotypes of 39 isolates from other Asian countries (*Brucella*2012MLVA database) were also included in this study to facilitate evaluation of genetic relationships with strains from neighboring countries.

## Results

### Identification of *Brucella* by AMOS-PCR and biotyping

All 81 *Brucella* strains were identified a*s B. melitensis* biovar 3 by conventional biotyping. The identification was confirmed by AMOS-PCR which, in accordance with our conventional biotyping, detected the amplified products (731 bp) of the strains.

### Typing and clustering of *B. melitensis* isolates by MLVA-16

The 81 *B. melitensis* biovar 3 isolates were clustered in 62 different genotypes, using the complete MLVA-16 assay (including panel 1 and the 2A and 2B loci). The corresponding diversity indexes (estimated by the HGDI) for panels 1, 2A, and 2B were 0.049, 0.096, and 0.992, respectively. The overall discriminatory index of MLVA-16 in this population was 0.992. Table 1 shows the diversity coefficient, the confidence interval, the number of alleles, and the fraction of samples that have the most repeat numbers in this locus. The bruce06, bruce08, bruce11, bruce12, bruce45, and bruce55 of panel 1 and bruce18 and bruce21 of panel 2A were completely homogeneous. In contrast, the most discriminatory markers were bruce04, bruce16, and bruce30 of panel2B (diversity index > 0.740), harboring 9, 9 and 8 alleles, respectively. The present population was clustered into two known genotypes and a new genotype by using panel 1. The two known genotypes were included in the previously recognized eastern Mediterranean group with genotypes 42 (79 strains) and 43 (1 strains). The new genotype (1-5-3-13-2-1-3-2) was a single-locus variant (SLV) to genotype 42 (1-5-3-13-2-2-3-2) which was recently numbered 114 in MLVAorsay genotypes [16].

**Table 1:** Polymorphism indexes of individual and combined VNTR loci in 81 *B. melitensis* biovar 3 isolates.^a^

**Panel and Locus**	**Diversity index^b^(HGDI)**	**Confidence interval^c^**	**K^d^**	**Max (pi)^e^**
**MLVA-16**	0.992	0.988–0.997	62	0.049
**Panel 1**	0.049	0.000–0.114	3	0.975
bruce06	0	0.000–0.085	1	1
bruce08	0	0.000–0.085	1	1
bruce11	0	0.000–0.085	1	1
bruce12	0	0.000–0.085	1	1
bruce42	0.025	0.000–0.072	2	0.988
bruce43	0.025	0.000–0.072	2	0.988
bruce45	0	0.000–0.085	1	1
bruce55	0	0.000–0.085	1	1
**Panel 2A**	0.096	0.009–0.184	3	0.951
bruce18	0	0.000–0.085	1	1
bruce19	0.096	0.009–0.184	3	0.951
bruce21	0	0.000–0.085	1	1
**Panel 2B**	0.992	0.987–0.997	61	0.049
bruce04(locus-6)	0.840	0.815–0.866	9	0.247
bruce07	0.338	0.218–0.458	3	0.802
bruce09(locus-8)	0.025	0.000–0.072	2	0.988
bruce16	0.842	0.812–0.873	9	0.259
bruce30(locus-2)	0.746	0.688–0.804	8	0.395
**HOOF-8**	0.999	0.998–1.000	79	0.025
locus-1	0.903	0.886–0.921	13	0.16
locus-3	0	0.000–0.085	1	1
locus-4	0.877	0.852–0.901	11	0.222
locus-5	0.902	0.877–0.928	14	0.198
locus-7	0.883	0.860–0.906	13	0.185

^a^Markers were studied by MLVA-16 and HOOF typing; n = 81.

^b^Hunter and Gaston index.

^c^Precision of the diversity index, expressed as 95% upper and lower boundaries.

^d^Number of different repeats at this locus or genotypes with combined loci.

^e^Fraction of samples that have the most frequent repeat numbers in this locus (range, 0.0 to 1.0).

**Figure 1:**
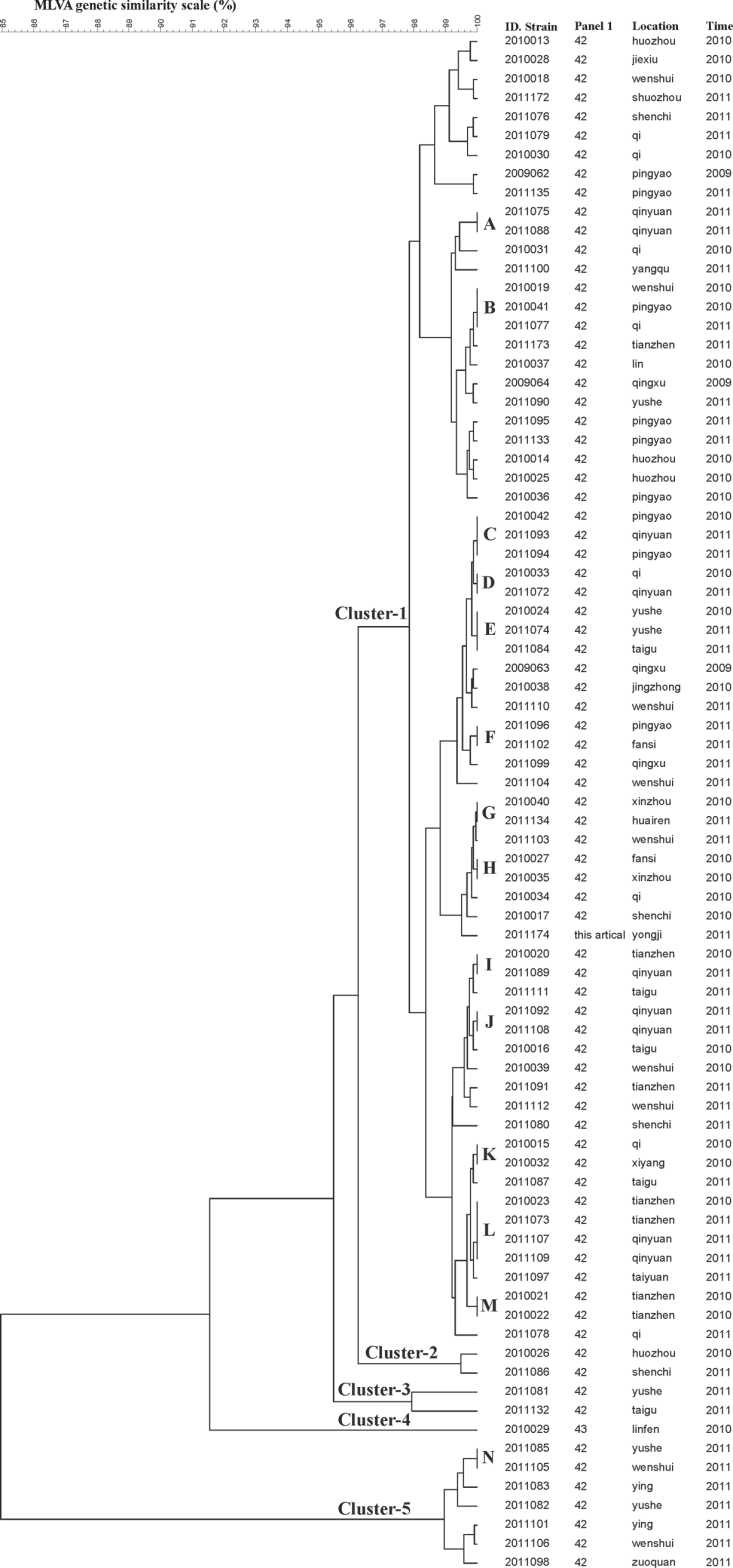
Dendrogram based on the MLVA-16 genotyping assay (UPGMA method), showing relationships between the 81 *B. melitensis* isolates. The columns show the identification numbers, the panel 1 genotypes, their geographic origin, and the year of isolation of the strains.

A dendrogram of the 81 *B. melitensis* biovar 3 strains which indicated the identification of the strains, the panel 1 genotype, and their geographical origins and year of isolation was stated (Fig. 1). The MLVA-16 assay allowed the population to cluster into 5 groups showing 84.95% similarity. For the different MLVA types, the maximum similarity of single locus variants (SLVs) and double locus variants (DLVs) was 99.89% and 99.79%, respectively.

**Figure 2:**
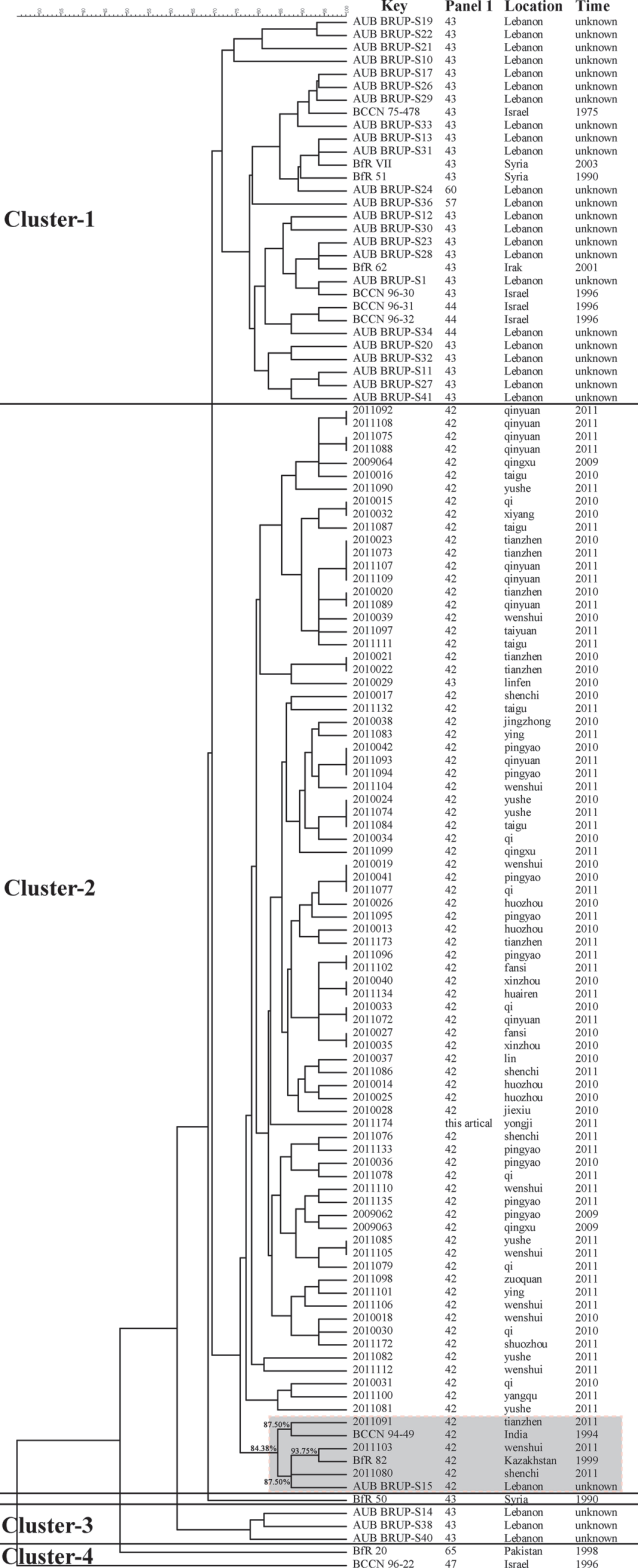
Dendrogram derived from the MLVA-16 genotyping assay (UPGMA method), showing relationships between the 81 Shanxi isolates and 39 Asian isolate. The columns show the identification numbers, the panel 1 genotypes, their geographic origin, and the year of isolation of the strains.

This analysis was extended to 39 isolates from other Asian countries (*Brucella*2012MLVA database) and the genetic relationship among these *B. melitensis* was determined (Fig. 2). The totals of 120 isolates were separated into four major clusters. At 71.69% similarity, cluster-1 included the eastern Mediterranean group with genotypes 43, 44, 57, and 60. There was 69.70% genetic similarity between genotype 43 from Shanxi (cluster 2) and genotype 43 from Lebanon (cluster 3). At 75.86% similarity, cluster-2 contained all 81 Shanxi isolates and 3 Asian isolates (India, Kazakhstan, and Lebanon) belonging to the eastern Mediterranean group, including genotype 42. At 75.86% similarity, strain 2011091 (from Tianzhen) and strain 2011080 (from Shenchi) formed a group with those 3 Asian isolates. Cluster-3 contained three isolates from Lebanon (genotype 43). Cluster-4 contained two American group genotypes, specifically 47 and 65.

### Correlation between MLVA-16 and HOOF-print results

**Figure 3:**
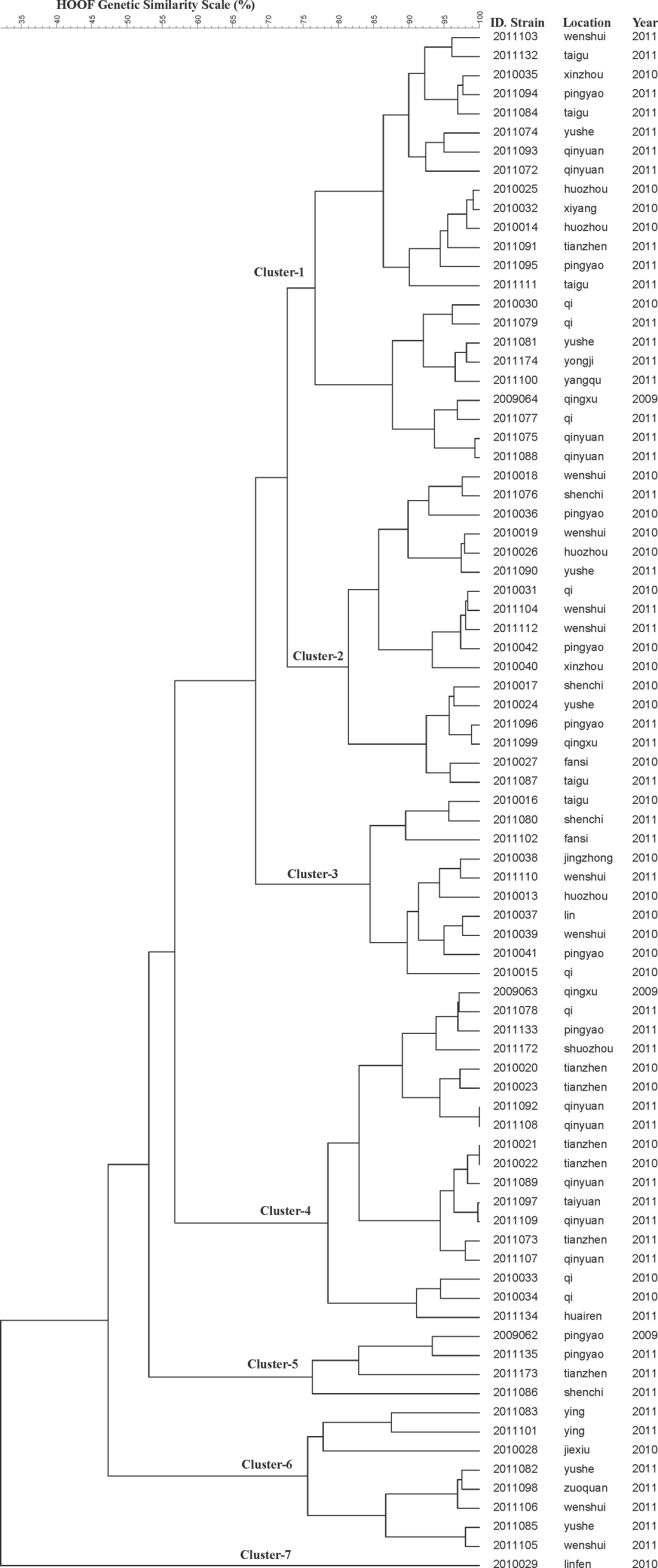
Dendrogram of HOOF types (UPGMA method), corresponding to the 81 *B. melitensis* strains from the Shanxi province. The columns show the identification numbers, years of isolation, and the geographic origins of the strains.

Seventy-nine different HOOF-prints were obtained for the present population, showing a diversity index of 0.999 with the confidence interval of 0.998 to 1.000. Table 1 shows the characteristics of each marker in the HOOF-print. The most discriminatory markers were locus-1 and locus-5 with a diversity index of > 0.900. In contrast, the most homogeneous marker was locus-3 with one allele. In the HOOF dendrogram, which was constructed with the UPGMA method (Fig. 3), the different HOOF types ranged in similarity from 32.08% and 100%. The HOOF-print typing allowed the population to cluster into 7 groups, showing 72.72% similarity. This increased the diversity recorder, with strains showing a smaller number of SLVs. Neither the MLVA-16 nor the HOOF-print genotype clusters corresponded to the specific county or year of isolation.

The HOOF-print produced 14 clusters with identical MLVA types, ranging in similarity from 56.79% to 100% (Table 2). It should be noted that the strains of sets J and M did not show diversity in the HOOF-print either and that they were isolated from the same county in the same year. Two SLVs of locus-5, repeats 8 and 7, belonged to set A. Four strains were DLVs of locus-4 and locus-7, repeats 5 and 6, from set L.

**Table 2:** Epidemiological and HOOF-print characteristics of clinical *B. melitensis* strains with identical MLVA types.^a^

**Set^b^ (n^c^)**	**Geographical location**	**Yr of isolation**	**HOOF-print VNTR with differences**	**Genetic similarity index(%)/variants^d^**
A (2)	Qinyuan	2011	5	99.37/SLVs
B (3)	Wenshui, Pingyao, Qi	2010, 2011	1,4,5,7	68.25–72.72
C (3)	Pingyao, Qinyuan	2010, 2011	1,4,5,7	72.72–89.98
D (2)	Qi, Qinyuan	2010, 2011	4,5,7	93.85
E (3)	Yushe, Taigu	2010, 2011	1,5,7	72.72–76.69
F (2)	Pingyao, Fansi	2011	1,4,5,7	68.25
G (2)	Xinzhou, Huairen	2010, 2011	1,4,5,7	56.79
H (2)	Fansi, Xinzhou	2010	1,5	72.72
I (2)	Tianzhen, Qinyuan	2010, 2011	1,4,7	82.92
J (2)	Qinyuan	2011	None	100
K (2)	Qi, Xiyang	2010	1,4,5,7	68.25
L (4)	Tianzhen, Qinyuan	2010,2011	1,4,7	82.92–98.00/ DLVs
M (2)	Tianzhen	2010	None	100
N (2)	Yushe, Wenshui	2011	1,5	98.03

^a^n = 33.

^b^Set referred to in MLVA-16 UPGMA dendrogram (see Fig. 1).

^c^n, no. of strains.

^d^Detection of SLVs and DLVs.

### Analysis of the 81 brucellosis cases

**Figure 4:**
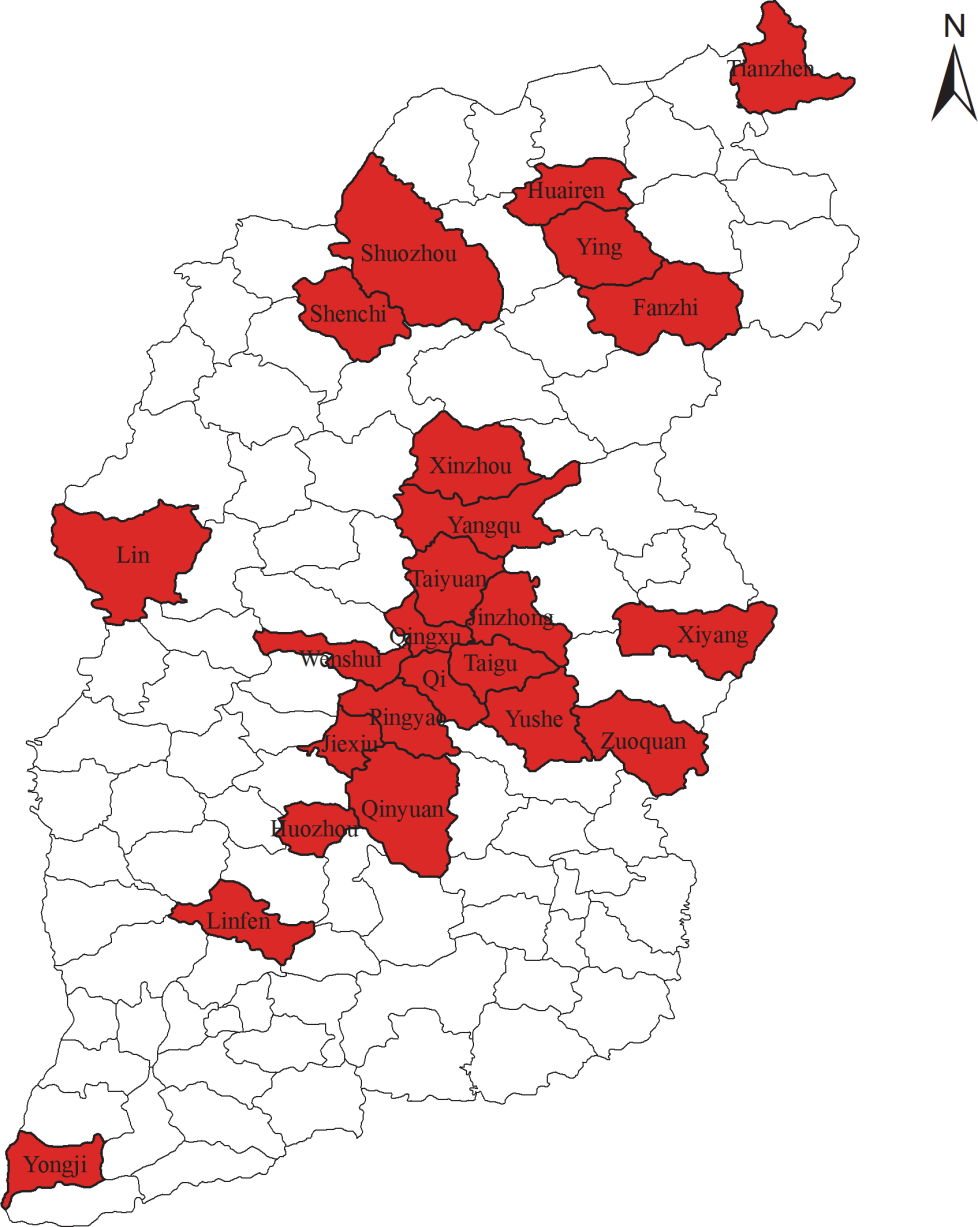
Distribution of *B. melitensis* isolates among 24 counties in Shanxi regions (marked in red).

All 81 *B. melitensis* biovar 3 isolates came from patients in only 24 counties within Shanxi Province. The geographical distribution of *B. melitensis* isolates in the Shanxi region is shown in Fig. 4. The majority (80.2%) of the brucellosis cases occurred between March and May. The number of male patients was almost 7 times that of female patients. The ages ranged from 14 to 73 years. The patients whose ages were between 23 and 60 years accounted for 82.7% of the total, and 77.8% of patients had contact with sheep. The relative number of cases with fever, joint pain, sweating, and fatigue were 67.9%, 39.5%, 16.0%, and 39.5%, respectively. Two cases showed orchitis (Table 3).

**Table 3:** Descriptive statistics of 81 brucellosis cases isolates in Shanxi province.

**Variable**		**N (%)**
Sex		
	male	70(86.42)
	female	11(13.58)
Age		
	<20	4(4.94)
	20–30	2(2.47)
	31–40	19(23.46)
	41–50	26(32.10)
	51–60	19(23.46)
	>60	11(13.58)
clinical symptoms		
	fever	68(83.95)
	fatigue	47(58.02)
	sweat	13(16.05)
	joint	44(54.32)
	orchitis	2(2.47)
Contact with sheep		
	yes	63(77.78)
	no	18(22.22)

### Epidemiological association between Shanxi and other provinces

**Figure 5:**
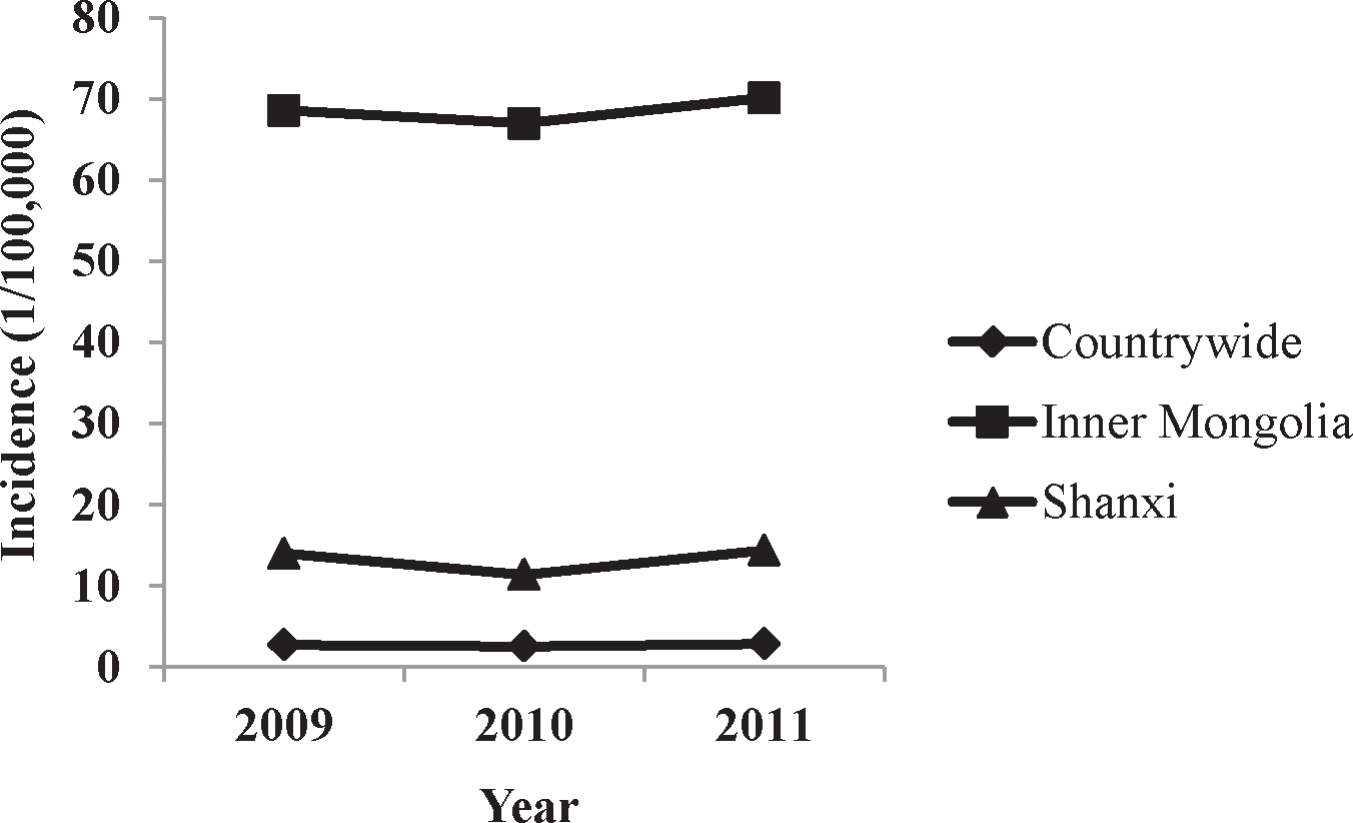
Line chart of the incidence of Shanxi and Inner Mongolia from 2009 to 2011.

Almost all the isolates from Shanxi belonged to the eastern Mediterranean group to the genotypes 42 (79 strains) and 43 (1 strain); this pattern was consistent with that of the isolates from Inner Mongolia [16]. The incidence of brucellosis in Shanxi was 14/100,000 cases in 2009, 11.4/100,000 in 2010, and 14.4/100,000 in 2011, while the incidence in Inner Mongolia was 68.6/100,000 in 2009, 67.0/100,000 in 2010, and 70.1/100,000 in 2011. Shanxi and Inner Mongolia were very similar regarding both the panel 1 genotypes of the isolates and the trend of incidence fluctuation (Fig. 5). The new genotype (1-5-3-13-2-1-3-2) found in this study was recently numbered 114 among MLVAorsay genotypes. It showed similarity to two isolates from Guangdong in 2008 in our earlier research [16]. These two patients from Guangdong had no at-risk occupational history and experienced fever and joint pain.

## Discussion

Before the 1980s, the endemic regions of brucellosis in China mainly involved areas with large amounts of pastureland, such as Inner Mongolia and Xinjiang. After the 1980s, the endemic regions gradually shifted to semi-agricultural and agricultural areas and small towns, particularly Shanxi and Liaoning. The incidence and frequency of outbreak of brucellosis have increased significantly in these regions. *B. melitensis* is the most predominant strain associated with the epidemic in China. Brucellosis season peaks from February to June [5, 17].

The incidence of brucellosis in Shanxi has made China’s top three among all Chinese provinces. All 81 clinical strains isolated from patients in the sentinel hospitals in Shanxi were collected as part of standard patient care between 2009 and 2011. These were identified as *B. melitensis* biovar 3. The results of this study coincide with the fact that, since 2005, most human cases in China have been caused by *B. melitensis* biovar 3 [16, 18]. They suggest that *B. melitensis* biovar 3 is the predominant strain associated with the epidemic of brucellosis in Shanxi Province. Sheep infected with *Brucella* are one of the main sources of infection for humans in Shanxi: 77.8% of cases had a confirmed history of exposure to sheep. Men seemed more likely to engage in slaughter, milking, and other work related to livestock. This exposure to livestock may account for the differences between males and females with regard to infection. Sexual behavior was not a relevant factor in the transmission of brucellosis because all patients were from different families. The remaining cases were in patients who denied having contact with living animals. These patients were mainly farmers, and a small number were urban residents. Among these patients, urban residents were retirees, housekeeping matrons, teachers, and unemployed patients. These findings indicate that even people without at-risk occupations can acquire brucellosis if they handle fresh meat or meat products for home cooking.

Most of the sentinel counties from which the strains were isolated were located in the middle of the province, as shown in Fig. 4. However, the incidence of brucellosis in northern Shanxi is higher than that in central and southern districts. This may be related to the good economic standing of northern Shanxi and its ability to support *Brucella* culture and enhanced laboratory monitoring. Another important reason is that underreporting phenomenon is serious in low-incidence areas. Considering differences in the submission of samples for testing among counties, there could also be submission bias within counties. Poor people are less likely to seek medical care than wealthier people. Due to sample collection by passive surveillance system, the sentinel collection of samples and potential submission bias may directly affect the spatial and temporal distribution patterns of brucellosis. The actual incidence of brucellosis in Shanxi Province may be partially reflected. To determine the epidemiological characteristics of *Brucella*, etiological surveillance work should be strengthened in the entire province.

Most of the isolated strains were from the eastern Mediterranean group, including genotype 42. This distribution pattern is consistent with that in Inner Mongolia [16]. The trend of the incidence fluctuation of the two provinces is consistent, with a similar pattern in the prevalence of animal brucellosis (unpublished observation). This result may be explained by the fact that Inner Mongolia is adjacent to northern Shanxi and there is a frequent livestock exchange between the two areas [17]. The new 114 genotype (MLVA8:1-5-3-13-2-1-3-2) found in Yongji in Shanxi was the same as two isolates from Guangdong found in 2008 (S1 Fig.). The HOOF-type of 114 Shanxi strain and the two 114 Guangdong strains is 8-5-1-11-8-3-6-3, 8-5-1-9-8-3-7-3, and 9-5-1-9-10-3-7-3 separately. This result may indicate the epidemiological association between Shanxi and Guangdong. Guangdong province, which is now considered endemic for brucellosis, is located in China’s southern coastal region, where the incidence of human brucellosis has increased gradually since 2000 [16]. Guangdong is an economically developed province. It has almost no livestock, and its meat products are mainly imported from Inner Mongolia and Shanxi. The new common genotype that was found both in Shanxi and Guangdong may indicate that livestock from Shanxi carried brucellosis from Shanxi to Guangdong and may thus pose an infection risk to people in Guangdong. Since 2000, the prevalence has been the highest in the eastern, central, and southern provinces in Shanxi, Hebei, Henan, and Guangdong. This suggests that the geographic distribution has also changed, with the disease traveling south. Inspection and quarantine of livestock exported from endemic regions needed to be strengthened. Species identification and subtyping of *Brucella* isolates is very important to epidemiologic surveillance and investigations of outbreaks in *Brucella*-endemic regions [3,4]. Genotyping methods such as MLVA and HOOF, which have improved the level of surveillance, have been used to distinguish strains that belong to the same biovar [8,10,11,19]. One of the aims of the present work was to examine the resolution of MLVA-16 and HOOF-print typing in discriminating between *B. melitensis* biovar 3 strains collected from 2009 to 2011 in Shanxi Province. In this study, both the complete MLVA-16 and HOOF-print provided very good discriminatory power, and the Hunter and Gaston indices were 0.992 and 0.999, respectively.

Unlike the HOOF-print, which, like MLVA-16, is tandem repeat-based; MLVA-16 has sixteen markers divided into complementary panels. The low diversities of panels 1 and 2A in this study (HGDI < 0.100) (Table 1) limited their role in the analysis of *B. melitensis* phylogeny [16, 20]. Analysis of *B. melitensis* phylogeny is useful to investigations of global geographical distributions. Cluster analysis of these strains isolated in the Shanxi Province, which is the epidemic focus of brucellosis in China, was based on the eight variable-nucleotide tandem repeat loci included in the MLVA-16 panel 1. The strains were classified as *B. melitensis* eastern Mediterranean group (panel 1, genotypes 42 and 43), except one strain which was newly found in this article in the Yongji county (1-5-3-13-2-1-3-2). Yongji is the southernmost county in the Shanxi Province, as shown in Fig. 4. Therefore, the high polymorphism of the panel 2B markers (HGDI = 0.992) and HOOF-print (HGDI = 0.999) benefited the surveillance of brucellosis in Shanxi Province. The genetic homogeneity of the Shanxi *B. melitensis* population with a genetic similarity ranging from 85% to 100% suggests recent evolution from a common ancestor in Shanxi. It is tempting to speculate that this may have been the predominant lineage in Shanxi. Genotype 42, as shown, is widely distributed throughout China and has previously been reported to be predominant in Turkey, Portugal, and Spain [19]. In China, *B. melitensis* genotype 42 strains were predominant in Inner Mongolia, Heilongjiang, Jilin, Hebei, and Shanxi provinces. These provinces are located in northern and eastern of China, where animal husbandry is the most important aspect of the economy. However, genotype 42 strains were reported in sporadic cases in other places, such as Liaoning, Shandong, Zhejiang, Fujian, and Tianjin. These isolates were only single-locus or double-locus variants of *B. melitensis* from the endemic regions [16].

When compared with Asian strains, only three strains (2011091, 2011103 and 2011080) showed the higher genetic similarity coefficients ranging from 87.50% to 93.75% with India, Lebanon, and Kazakhstan strains. This might suggest that poor importation quarantine policies may account for a subset of *B. melitensis* infections. Together, these data suggest that the MLVA-16 assay can be applied to long-term surveillance and investigations of *B. melitensis* origins and epidemiology.

Half of the markers required for MLVA-16 and HOOF-printing showed a higher degree of diversity among the *B. melitensis* biovar 3 strains in this study than in other studies. There were only two pairs of strains with 100% genetic similarity based on HOOF-printing. Two strains from Qinyuan County that were isolated in 2011 had the HOOF genotype 8-4-1-7-3-5-10-3. Another two strains from Tianzhen, isolated in 2010, had the HOOF genotype 11-4-1-10-2-8-8-3. The two patients from Tianzhen both lived in the southern part of the county but they were not from the same village. The two patients from Qinyuan were from the same village in the same county. The results revealed that these patients may have had a common exposure history. Some apparently unlinked (epidemiologically or otherwise) isolates also had identical MLVA-16 profiles. More detailed genetic studies, such as whole-genome sequence comparisons must be carried out to confirm these relationships and strain microevolution.

Both MLVA-16 and HOOF-printing, which are based on tandem repeats, showed an epidemiological relationship between *B. melitensis* biovar 3 populations in the endemic region. These techniques are very promising ways of investigating strain relatedness in regions of endemicity [21–24]. Shanxi isolates formed a homogeneous group and appeared to be most closely related to some human *B. melitensis* in the eastern Mediterranean group. Abbreviated MLVA schemes (omitting testing with panels 1 and 2A) or HOOF-printing in regions of endemicity may facilitate investigation of outbreaks. In areas not endemicity for brucellosis, the MLVA-16 scheme is very helpful to prevent the expansion and eradicate the disease. Interestingly, neither the MLVA-16 nor the HOOF-print genotype clusters corresponded to the specific county or year of isolation. There are some possible reasons for this phenomenon. First, homoplasy levels at the most variable loci may be responsible for this, illustrating the importance of marker selection. Second, any isolates with a distinct genotype might cause only sporadic cases. Third, there is a diverse mixture of strains in the animal population in the restricted area or human-infected *Brucella* strains originated from different regions through trade of animal products. Finally, the existing passive surveillance program showed some unavoidable shortcomings. More isolates are needed to further consolidate this finding.

Some European countries have carried out veterinary policies to restrict the brucellosis infection in flocks, with benefits in human public health [25, 26]. The situation of brucellosis in certain provinces within China is rapidly worsening. Increasing numbers of cases of brucellosis have shown that the strategy of vaccination and quarantine for infected animals has failed in China. One possible reason is the limited efficacy of the current vaccines [27, 28]. Another reason is that the policies for eradication and control of *Brucella*-infected animals and their products may not have been adequately implemented [29]. Awareness of the epidemiological map of human brucellosis may allow inter-province public-health organizations to take proper interventions. There is an immediate need for a concerted effort to control and eradicate brucellosis transmitted through domesticated animals in China.

## Supporting Information

S1 Fig Distribution of Panel 1 genotypes of the strains isolated in Shanxi, Inner Mongolia, and Guangdong provinces. Triangle, rectangle, asterisk represent 42, 43, and 114 Panel 1 genotypes respectively.
(TIF)

S1 Table MLVA-16 genotypes for 120 *B. melitensis* strains.
(XLS)

S2 Table HOOF genotypes for 81 *B. melitensis* strains.
(XLS)
