# Supplementary figures and images for: Genotyping of Human Brucella melitensis Biovar 3 Isolated from Shanxi Province in China by MLVA16 and HOOF
Source: PLoS One. 2015 Jan 23;10(1):e0115932. doi: 10.1371/journal.pone.0115932 (PMC4304826; doi:10.1371/journal.pone.0115932)

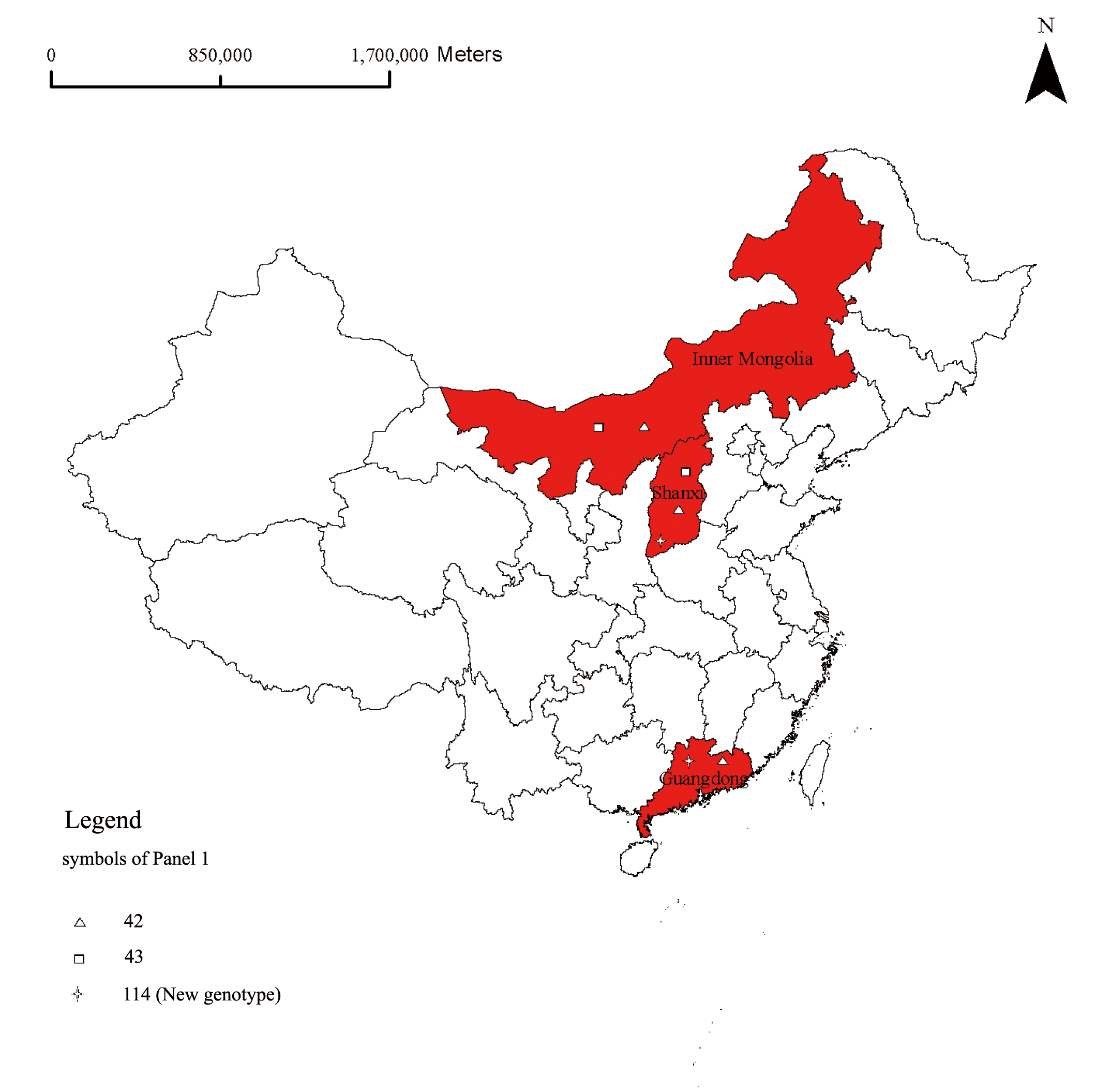

Supplement: S1 Fig — Triangle, rectangle, asterisk represent 42, 43, and 114 Panel 1 genotypes respectively. (TIF) [file pone.0115932.s001.tif]
